# Isolated Phlegmon of the Round Ligament of the Liver: Clinical Decision-Making in the Context of Lemmel’s Syndrome—A Case Report

**DOI:** 10.3390/reports8040192

**Published:** 2025-09-29

**Authors:** Georgi Popivanov, Marina Konaktchieva, Roberto Cirocchi, Desislava Videva, Ventsislav Mutafchiyski

**Affiliations:** 1 Department of Surgery, Military Medical Academy, 1606 Sofia, Bulgaria; 2 Department of Gastroenterology and Hepatology, Military Medical Academy, 1606 Sofia, Bulgaria; 3 Department of Digestive and Emergency Surgery, “S. Maria” Hospital, 05100 Terni, Italy; 4 UHAT “Sv. Ivan Rilski”, 1431 Sofia, Bulgaria

**Keywords:** round liver ligament, phlegmon, duodenal diverticulum, Lemmel’s syndrome

## Abstract

**Background and Clinical Significance**: The pathology of the round ligament (RL) is rare and often remains in the shadow of common surgical emergencies. The preoperative diagnosis is challenging, leaving the surgeon perplexed as to whether and when to operate. The presented case deserves attention due to the difficult decision to operate based solely on the clinical picture, despite negative imaging diagnostic results. **Case presentation:** A 76-year-old woman was admitted to the Emergency Department with 6 h complaints of epigastric pain, nausea, and vomiting. She was afebrile with stable vital signs. The abdomen was slightly tender in the epigastrium, without rebound tenderness or guarding. The following blood variables were beyond the normal range: WBC—13.5 × 109/L; total bilirubin 26 mmol/L; amylase—594 U/L; CRP 11.4 mg/L; ASAT—158 U/L; and ALAT—95 U/L. The ultrasound (US) and multislice computed tomography (MSCT) of the abdomen were normal. A working diagnosis of acute pancreatitis was established, and intravenous infusions were initiated. The next day, the patient became hemodynamically unstable with blood pressure 80/60 mm Hg, heart rate 130/min, chills and fever of 39.5 °C, and oliguria. There was remarkable guarding and rebound tenderness in the epigastrium. The blood analysis revealed the following: WBC—9.9 × 109/L; total bilirubin—76 µmol/L; direct bilirubin—52 µmol/L; amylase—214 U/L; CRP 245 mg/L; ASAT—161 U/L; ALAT—132 U/L; GGT—272 U/L; urea—15.7 mmol/L; and creatinine—2.77 mg/dL. She was taken to the operating room for exploration, which revealed local peritonitis and phlegmon of the RL. Resection of the RL was performed. The microbiological analysis showed Klebsiella varicola. The patient had an uneventful recovery and was discharged on the 5th postoperative day. In the next months, the patients had several readmissions due to mild cholestasis and pancreatitis. The magnetic resonance demonstrated a duodenal diverticulum adjacent to the papilla, located near the junction of the common bile and pancreatic duct. This clinical manifestation and the location of the diverticulum were suggestive of Lemmel’s syndrome, but a papillary dysfunction attributed to the diverticulum or food stasis cannot be excluded. **Conclusion:** To our knowledge, we report the first association between RL gangrene and Lemmel’s syndrome. We speculate that duodenal diverticulitis with lymphatic spread of the infection or transient bacteriemia in the bile with bacterial translocation due to papillary dysfunction, as well as cholestasis resulting from the diverticulum, could be plausible and unreported causes of the RL infection. The preoperative diagnosis of RL gangrene is challenging because it resembles the most common emergency conditions in the upper abdomen. The present case warrants attention due to the difficult decision to operate based solely on the clinical picture, despite negative imaging results. A high index of suspicion should be maintained in a case of unexplained septic shock and epigastric tenderness, even in negative imaging findings. MSCT, however, is a valuable tool to avert unnecessary operations in conditions that must be managed conservatively, such as acute pancreatitis.

## 1. Introduction and Clinical Significance

Emergency general surgery (EGS) carries a substantial healthcare burden. Although EGS patients account for only 7% of hospital admissions worldwide, the direct costs amount to $28 billion, which is more than for other emergency conditions [1]. The emergency general surgeon has to manage various emergency conditions. Defining the scope of EGS, Shafi et al. identified 96 ICD-9 diagnoses that may present to the emergency surgeon, underscoring the need for specific training and research [2]. M. Shein compares emergency surgery with the infantry—“the war cannot be won without infantry on the ground, … in the emergency department it is the patient and you—you who are bound to provide a correct management plan and execute it” [3]. The proper plan, however, is hampered in rare conditions mimicking a wide variety of pathologies in the upper abdomen, which makes the preoperative diagnosis challenging and leaves the surgeon perplexed as to whether and when to operate. The round ligament (RL) pathology is rare and usually remains in the shadow of the common surgical emergencies. Up to 2021, only 44 cases of isolated inflammation, gangrene, or abscess of the RL were published worldwide, one of which was managed in our Institution in 2002 [4,5,6].

The present case warrants attention due to the challenging decision to operate based solely on the clinical picture, despite negative imaging diagnostic results (X-ray, ultrasound (US), and multislice computed tomography (MSCT). Moreover, it probably represents the first association of RL phlegmon with Lemmel’s syndrome.

## 2. Case Presentation

A 76-year-old woman was admitted to the Emergency Department with 6 h complaints of epigastric pain, nausea, and vomiting. The previous medical history included arterial hypertension, stable ischemic heart disease, type 2 diabetes, removal of the right ovary, and open onlay mesh repair of postoperative hernia. The BMI was 30. She was afebrile with a blood pressure of 110/70 mm Hg and a heart rate of 70/min. The abdomen was slightly tender in the epigastrium, without rebound tenderness or guarding; normal bowel sounds were present. The blood variables at admission and day 1 are shown in Table 1.

**Figure 1:**
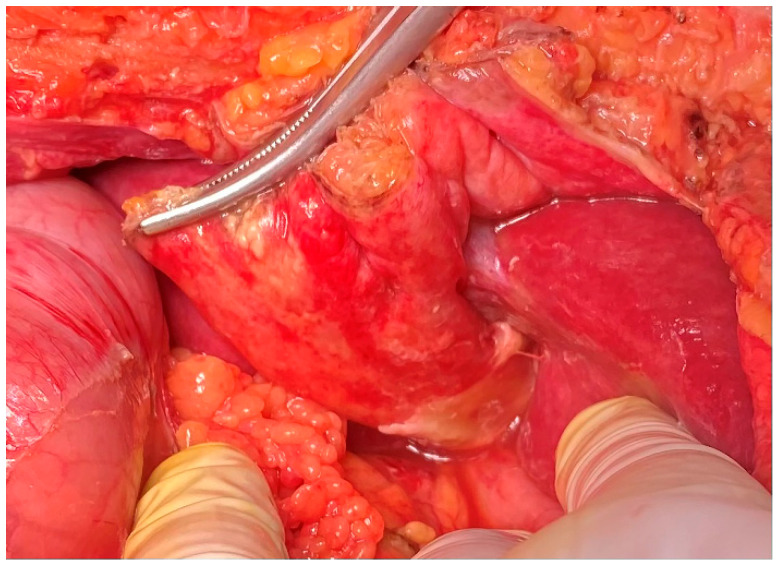
An intraoperative view of the inflamed RL with fibrin and ascites.

The ultrasound and CT of the abdomen were normal; there was neither free fluid nor free air in the abdomen. A working diagnosis of acute pancreatitis was established and intravenous infusions were initiated. The next day, a significant deterioration was observed. The patient became hemodynamically unstable with BP 80/60 mm Hg, HR 130/min, chills and fever of 39.5 °C, and oliguria. There was remarkable guarding and rebound tenderness in the epigastrium. The blood analysis revealed a sharp increase in CRP, total bilirubin, urea, creatinine, IgE and IL-6 (Table 1). The repeated ultrasound was normal. Given the rapid worsening of the general condition and the signs of peritonitis, she was taken to the operating room for exploration. An open laparoscopy was attempted, but it was unsuccessful due to massive adhesions from the previous operations. The midline upper laparotomy revealed local peritonitis and an inflamed round ligament covered with fibrin (Figure 1).

**Figure 2:**
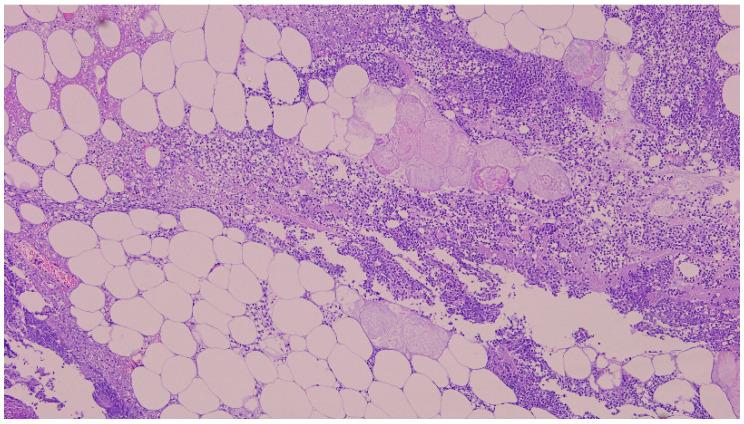
The histopathological examination of the resected surgical specimen (H&E) showed polymorphonuclear infiltration, steatonecrosis, fibrin, and foamy histiocytes (5×).

There were no signs of perforation of the hollow viscus organs, and the pancreas was observed to be normal. Resection of the RL was performed. The microbiological analysis of the biopsy sample revealed that Klebsiella varicola was susceptible to most antibiotics (MALDI-TOF MS, Bruker Corp., Billerica, MA, USA). The histopathological examination of the resected surgical specimen reveals a massive polymorphonuclear infiltration, diffuse steatonecrosis, and associated foamy histiocytes, as well as foci of fibrin and haemorrhage (Figure 2). The patient was treated with IV fluids, Piperacillin/Tazobactam, and PPI. She had an uneventful recovery and was discharged on the 5th postoperative day.

**Figure 3:**
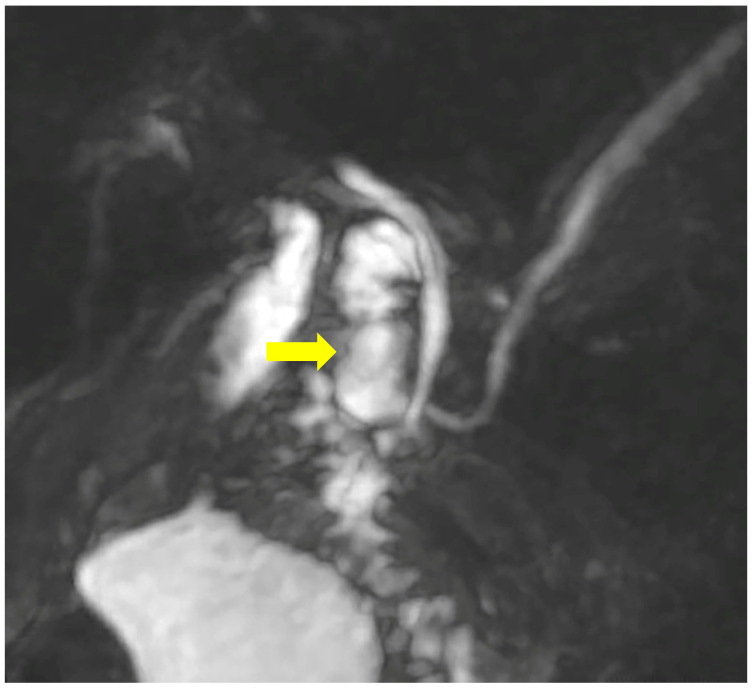
MRCP demonstrates the duodenal diverticulum over the junction of the biliary and pancreatic ducts (arrow).

**Figure 4:**
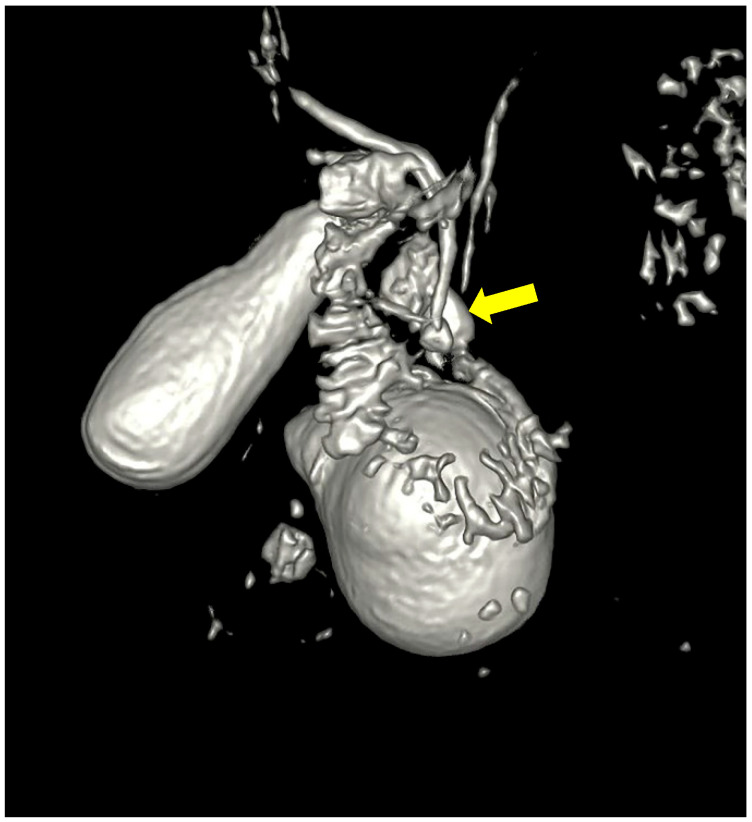
Three-dimensional reconstruction showing the duodenal diverticulum near the pancreatico-billiary junction (arrow).

A month later, the patient was readmitted due to upper abdominal pain, a slight elevation of transaminases, alkaline phosphatase, GGT, and amylase. CRP was within a normal range, with unremarkable US/MSCT findings. The magnetic resonance imaging (MRI) demonstrated a duodenal diverticulum adjacent to the papilla, located over the junction of the common bile and pancreatic ducts, which were not dilated (Figure 3 and Figure 4).

Esophagogastroduodenoscopy confirmed a juxta-papillary diverticulum without remarkable pathology. In the next four months, the patients suffered from four other attacks with abdominal pain and a slight and transient increase in bilirubin, transaminases, and amylase. Because all known causes for pancreatitis were excluded, a dysfunction of the papilla due to a diverticulum was hypothesised, and the patient was referred for ERCP in case of relapse.

## 3. Discussion

The RL represents a remnant of the obliterated umbilical vein, which connects the liver to the anterior abdominal wall. It may be affected by various pathologic conditions such as congenital defects, cysts, tumours, spontaneous rupture of portocaval shunts, and focal fat infarction [4,7]. RL may be secondarily inflamed in peritonitis, but the primary inflammation (gangrene) is extremely rare. Following the review of Bhatt et al. (44 cases), we identified four additional cases up to 2024 [8,9,10,11]. More important than the rarity, however, is how the emergency surgeon must make the correct diagnosis and decision to operate. Because of its rarity, RL gangrene is usually far beyond the sight of the emergency surgeon in the differential diagnostic plan. In most cases, the clinical manifestations overlap with those of acute peritonitis, mimicking the most common surgical emergencies in the upper abdomen, including cholecystitis, followed by cholangitis, acute pancreatitis, perforated duodenal ulcer, and intra-abdominal abscess [4,6]. In our opinion, the most challenging task is to distinguish RL gangrene from acute pancreatitis, which must be managed conservatively because early surgery significantly worsens the prognosis. Two cases in the review of Nolasco Vaz, however, have been associated with acute pancreatitis (Ito, 2008, 2013), with favourable outcomes [4].

In two-thirds of cases, the condition is characterised by sudden epigastric or right upper quadrant pain, fever, and nausea. An elevation of WBCs and CRP is observed in 75% [4]. In 25% of the cases, it may present with septic shock, as in the two cases from our institution [4,5]. Our analysis of the review of Nolasco Vaz et al. and the new cases demonstrated an isolated pathology of RL in 46% of the cases [4,8,9,10,11]. MSCT typically reveals heterogeneous, hypodense RL, adjacent fat stranding or abscess, but in fact, it allows a correct diagnosis in only 40% of the cases [4,12,13,14]. The most common wrong diagnoses were biliary pathology, acute pancreatitis, and intra-abdominal abscesses [4,6]. One reason could be the rarity of the disease and the lack of awareness, as seen in the case of Bhatt et al., where a postoperative review of the MSCT confirmed the diagnosis [6]. Nevertheless, it is helpful to exclude other emergency abdominal conditions. It is crucial to exclude acute pancreatitis, where the untimely operation may lead to an adverse outcome. MRI could be helpful for diagnosis, especially in cases with concomitant biliary pathology or small abscesses appearing as a mass with disseminated foci in the liver [8]. Sen et al. demonstrated an abscess of the RL appearing as a sausage-like cystic lesion on MRI and synchronous choledocholithiasis [13]. Kuribara et al. mass with small high-intensity areas on T2-weighted images [8]. MRI, however, is limited in emergency settings.

The conservative treatment has a 50% failure rate [4]. The vast majority of cases were treated surgically despite the successful antibiotic treatment in some reports [4,9]. In the presented case, we undertook surgery because of the significant deterioration of the condition and the signs of peritonitis despite the negative imaging findings. Due to the uncertain diagnosis, taking into account the possibility of a wrong decision in case of unrecognised acute pancreatitis, we attempted diagnostic laparoscopy, which was unsuccessful due to massive adhesions from the previous operations. Laparoscopy is not only a feasible therapeutic option, but also a useful minimally invasive diagnostic tool in case of diagnostic uncertainty [4].

The pathophysiology of RL gangrene is still poorly understood, and we can only speculate about the real cause. Understanding the ligament’s blood supply is crucial because the two plausible hypotheses include ischemic events or hematogenous/lymphatic spread of the infection. The RL is supplied by superior and inferior groups of vessels [15,16]. The superior arterial vessels are terminal branches of the internal thoracic artery and the inferior phrenic arteries. The superior venous supply is similar. The inferior vessels originate from the right or left hepatic artery. Multiple small veins entering the umbilical and paraumbilical veins provide the inferior venous flow. Both superior and inferior vessels also anastomose with the intrahepatic vessels. The ligament has a dense network of lymphatic capillaries, which are interconnected with the superficial hepatic lymphatic system, the lymph nodes in the porta hepatis and mediastinum, the abdominal wall, the parasternal space, and also the free peritoneal cavity [17,18].

Given the caliber of the arteries is small, in case of insufficient collaterals, thrombosis of the main vessels may cause ischemia and necrosis [5,6]. On the other hand, the complexity of the vascular network of the RL implies that all pathologic conditions in the abdominal and thoracic wall, liver, and retroperitoneum may lead to inflammation of the RL. Kuribara et al. reported a new case of RL abscess with disseminated intrahepatic foci managed by liver resection, which demonstrates that the infection can spread to both near and distant organs [8].

In 51% of the cases, the RL gangrene could be related to biliary origin [4]. In one case, the same bacteria were isolated from the bile and the pus. The underlying mechanism is unclear, but a possible explanation is the bacterial translocation and lymphatic spread. Another possible route for a contiguous spread of the infection is through an aberrant bile duct in the RL, as in the case of Nolasco Vaz et al. [4]. Zorgdrager et al. described a case of a large abscess formation in the periumbilical abdominal wall [19]. The causal relationship in their case is a duodenal ulcer penetrating the bile duct, leading to subsequent cholangitis, phlebitis of the left portal vein, and dissemination of infection through the RL into the periumbilical area. Two patients have been reported with a previous hernia repair, as in our case (possible lymphatic spread) [6,20]. In all three cases, however, there was no history of mesh infection. Portal vein thrombosis is reported in 15% of cases, suggesting thrombosis of the small vessels of the RL [4]. This is also a possible mechanism in patients with atherosclerosis and diabetes, as in our case.

In the present case, the clinical manifestation and the location of the diverticulum were suggestive of Lemmel’s syndrome despite the absence of bile duct dilatation. Most papers reported a prevalence of duodenal diverticula between 10% and 22%, but only 1–5% are symptomatic [21,22,23]. Approximately 60% are located in the second portion of the duodenum, and almost all arise from the pancreatic border [21]. The clinical presentation varies from asymptomatic cholestasis and mild pancreatitis to complications such as perforation, bleeding, diverticulitis, or compression of the adjacent structures with jaundice, cholangitis, and pancreatitis [21,23]. The compression with dilatation of the common bile duct, known as Lemmel’s syndrome, was described by Gerhard Lemmel in 1934. The presenting symptoms of this rare condition resemble the common biliary pathology with upper abdominal pain, fever up to 38.5 °C, abdominal tenderness in the RUQ, jaundice, and moderately elevated transaminases. A dilated common bile duct is present in 100% of the cases [24]. We speculate that duodenal diverticulitis with lymphatic spread of the infection or transient bacteriemia in the bile with bacterial translocation due to papillary dysfunction, and cholestasis resulting from the diverticulum could be plausible and unreported causes of the RL infection. A food stasis and compression from the diverticulum or sphincter Oddi dysfunction could also explain the observed relapses of cholestasis and mild pancreatitis.

## 4. Conclusions

To our knowledge, we report the first association between RL gangrene and Lemmel’s syndrome. We speculate that duodenal diverticulitis with lymphatic spread of the infection through the above-mentioned network or transient bacteriemia in the bile with bacterial translocation due to papillary dysfunction, as well as cholestasis resulting from the diverticulum, could be plausible and unreported causes of the RL infection.

The preoperative diagnosis of RL gangrene is challenging because it resembles the most common emergency conditions in the upper abdomen. The present case warrants attention due to the difficult decision to operate based solely on the clinical picture, despite negative imaging results. A high index of suspicion should be maintained in a case of unexplained septic shock and epigastric tenderness, even in negative imaging findings. MSCT, however, is a valuable tool to avert unnecessary operations in conditions that must be managed conservatively, such as acute pancreatitis. MRI could be helpful for diagnosis, especially in cases with concomitant biliary pathology or small abscesses appearing like a mass with disseminated foci in the liver.

## Figures and Tables

**Table 1:** The laboratory values.

Laboratory Value	at Admission	Day 1	Reference Range
WBCs	13.5 × 10^9^/L	9.9 × 10^9^/L	3.5–10.5
Granulocytes	76%	89.4%	34–71
CRP	11.4 mg/L	245 mg/L	0–5
Total bilirubin	26 µmol/L	76 µmol/L	
Direct bilirubin		52 µmol/L	
ASAT	158 U/L	161 U/L	5–40
ALAT	95 U/L	132 U/L	5–40
GGT		272 U/L	10–50
Amylase	594 U/L	214 U/L	30–100
Urea	13.4 mmol/L	15.7 mmol/L	2.8–8.3
Creatinine	126 µmol/L	245 µmol/L	58–100
IgE		294.6 UI/ml	0–25
IL-6		13.43 pg/ml	5.3–7.5

## Data Availability

The data presented in this study are available on request from the corresponding author due to reasonable request.

## Abbreviations

EGS—Emergency general surgery, RL—round ligament, US—ultrasound, MSCT—multislice computed tomography, MRI—magnetic resonance imaging.
